# Cryo-electron tomography to study viral infection

**DOI:** 10.1042/BST20230103

**Published:** 2023-08-10

**Authors:** Miles Graham, Peijun Zhang

**Affiliations:** 1 Division of Structural Biology, Wellcome Trust Centre for Human Genetics, University of Oxford, Oxford OX3 7BN, U.K.; 2 Diamond Light Source, Harwell Science and Innovation Campus, Didcot OX11 0DE, U.K.; 3 Chinese Academy of Medical Sciences Oxford Institute, University of Oxford, Oxford OX3 7BN, U.K.

**Keywords:** correlative microscopy, cryo-electron tomography, cryo-EM, *in situ*, subtomogram averaging, virus infection

## Abstract

Developments in cryo-electron microscopy (cryo-EM) have been interwoven with the study of viruses ever since its first applications to biological systems. Following the success of single particle cryo-EM in the last decade, cryo-electron tomography (cryo-ET) is now rapidly maturing as a technology and catalysing great advancement in structural virology as its application broadens. In this review, we provide an overview of the use of cryo-ET to study viral infection biology, discussing the key workflows and strategies used in the field. We highlight the vast body of studies performed on purified viruses and virus-like particles (VLPs), as well as discussing how cryo-ET can characterise host–virus interactions and membrane fusion events. We further discuss the importance of *in situ* cellular imaging in revealing previously unattainable details of infection and highlight the need for validation of high-resolution findings from purified *ex situ* systems. We give perspectives for future developments to achieve the full potential of cryo-ET to characterise the molecular processes of viral infection.

## Introduction

A rigorous understanding of viral infection is essential to the development of new vaccines and antivirals to safeguard human health. While such developments have helped in bringing a close to the COVID-19 pandemic which has caused ∼5.42 million reported deaths, we remain vulnerable to SARS-CoV-2 and a plethora of other viruses [1]. In just the last decade, we witnessed thousands of deaths resulting from Ebola virus outbreaks and the occurrence of severe birth-defects as Zika virus rapidly spread worldwide [2,3]. Continued work to understand viral infection is therefore required to lay the foundations for rapid responses to future outbreaks of both known and novel viruses alike.

The study of viral structures by electron microscopy dates back to the very first three-dimensional (3D) reconstruction from two-dimensional (2D) projections using negative-stain electron microscopy 55 years ago [4,5]. Similarly, cryogenic sample preparation was initially demonstrated for viruses and the first high-resolution structures of icosahedral viruses by single particle cryo-EM later followed [6–8]. This approach has been highly successful for studying purified symmetric viral structures such as icosahedral or helical capsids [9–15]. However, single particle cryo-EM is limited in the capture of pleomorphic viral structures and unsuited to studying viral infection in the cellular context. In contrast, cryo-ET is now able to expand the reach of electron microscopy to more challenging targets and into infected cells themselves. In this review, we outline and review cryo-ET approaches to study viral infection. Particular focus is given to workflows which permit structural dissection of viral life cycles *in situ*, such as the use of cryo-CLEM (correlative light electron microscopy) and cryo-FIB/SEM (cryo-focussed ion beam milling with scanning electron microscopy) prior to cryo-ET with subtomogram averaging (cryo-ET STA).

## Overview of cryo-ET and adjacent methods

Cryo-ET requires a series of 2D projections to be captured with the microscope stage tilted through a range of angles with respect to the incident beam. These projections collectively are referred to as the ‘tilt-series' and represent different views of the target region. Once these projection images are motion-corrected and aligned with respect to one another using various software packages, they are used to generate tomograms which show a 3D volume of the target region, revealing the biological specimens contained within [16–19]. A collection of tomograms, or on occasion individual tomograms themselves, can allow for extensive interpretation of biological processes. However, in order to solve the structures of molecular complexes by cryo-ET, STA is required. Here, smaller volumes are extracted from tomograms, aligned and averaged to increase the signal-to-noise ratio of the selected feature, such as a viral protein or ribonucleoprotein complex (RNP) [20]. In this way, each particle exists as an individual 3D reconstruction, allowing for 3D variance between particles to be analysed. This makes cryo-ET STA uniquely suited to the study of heterogeneous and pleomorphic viral assemblies to high resolution.

**Figure 1.**
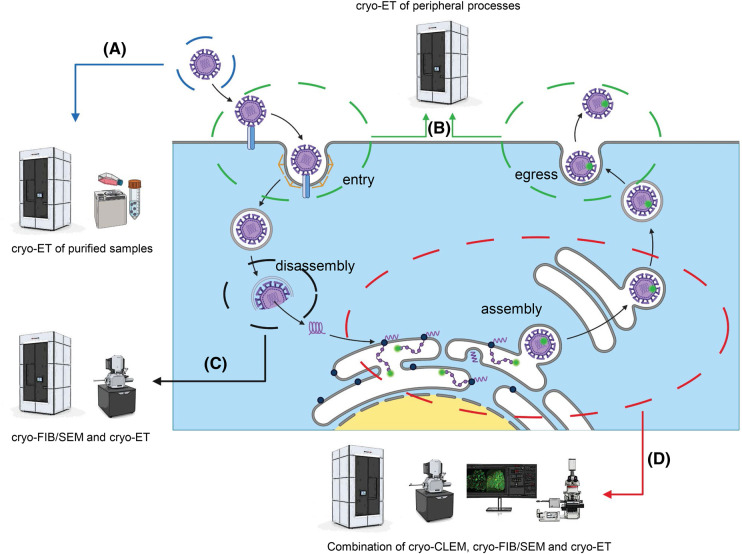
Workflows to study viral infection using cryo-ET. Schematic showing the common workflows for studying viral infection, put in the context of an example viral replication cycle of flavivirus which includes viral entry, disassembly and genome release, viral assembly and viral egress. (**A**) Purified and concentrated samples of virus can be studied directly using cryo-ET to allow high-resolution reconstructions. (**B**) Some viral infection processes which occur in the cell periphery are accessible to cryo-ET without sample thinning. (**C**) cryoFIB-SEM allows cryo-ET to examine processes occurring deeper in the cell. Milling is performed ‘blind' in the absence of correlative methods. (**D**) Cryo-CLEM permits targeting of the milling process to regions of interest (in this example an endogenously tagged viral protein seen in the green channel). After milling, cryo-ET is conducted as normal. Figure created with BioRender and is not to scale [29].

To collect useful tomograms and certainly to obtain higher resolution EM maps, the samples should be thin enough to allow sufficient penetration of the electron beam. Cryo-FIB/SEM is a powerful and rapidly developing technique to locally thin the sample while retaining high-resolution information for tilt-series acquisition in the selected regions [21]. Using a focused ion beam such as gallium, argon or xenon, the sample can be ‘milled' down as layers are ablated by the ion beam [22,23]. Thinned regions referred to as lamellae are created to provide windows through which high-resolution data may be collected. This opens up the possibility to structurally dissect viral infection processes happening within cells. Cryo-FIB/SEM is ideally combined with cryo-CLEM in order to signpost suitable locations in the sample at which to conduct the milling process [24]. Here, fluorescently tagged viral proteins or interacting host proteins allow the visualisation of infection processes in live cells using light microscopy, which once correlated with EM become markers at which cryo-FIB/SEM and cryo-ET may be conducted. Complementary modes of studying infected whole cells, albeit at lower resolutions, are the use of soft x-ray tomography (SXT) and serial cryo-FIB/SEM volume imaging [25–28]. Combinations of these methodologies allow highly challenging biological questions to be addressed across multiple scales. An overview of the common workflows for studying viral infection is presented in Figure 1 [29].

## Cryo-ET of purified viruses, virus-like particles (VLPs) and viral components

Two decades ago, the initial visualisation of viruses by cryo-ET relied on preparation of isolated virus, a method which remains central to the contemporary study of viral structure [30]. When imaging purified and concentrated viruses or VLPs, there is little concern about sample thickness or abundance of particles of interest. It is therefore unsurprising that this approach represents the most straightforward path to obtaining high-resolution structures by cryo-ET STA. Purified HIV-1 virus (or indeed retroviruses more broadly) and VLPs have been relentlessly studied for many years by cryo-ET STA [31–33], resulting in an exquisitely detailed structural understanding of the virus. The assembly of HIV-1 at the plasma membrane is driven by the Gag polyprotein and culminates in the budding of an immature virion exhibiting a Gag lattice associated with the viral membrane. Dramatic structural rearrangements then follow after Gag cleavage by the viral protease, to form the mature virion with the signature conical capsid containing RNP [34–36]. The technological developments of cryo-ET have permitted STA of the Gag lattice from purified immature viruses and VLPs to near-atomic resolution [37–41]. With a map resolved to 3.9 Å, Schur *et al.*were able to better understand the role of the capsid-SP1 (CA-SP1) region of Gag in maturation and identify how maturation inhibitors stabilise the immature lattice to restrict protease action [40]. Their study sensibly examined both immature virus particles and *in vitro* assembled Gag lattices to verify that there were no structural differences between the two, meaning that the higher resolution map of the latter could be used for valid interpretation. This dataset (EMPIAR-10164) is commonly used for benchmarking developments in image processing [42–47]. Advances over recent years mean that the immature Gag lattice shown here can now be visualised to 3.0 Å resolution. This is hypothesised to be the theoretical resolution limit, owing to the flexible, asymmetric nature of the CA hexamer and perhaps the data acquisition parameters [45,47]. The structural details of the matrix (MA) domain of HIV-1 Gag in both mature and immature configurations were recently revealed [48]. MA undergoes significant structural changes during maturation to form a lattice structure with different packing interactions, analogous to the immature and mature CA [48]. Improved understanding of MA organisation may shed more light on Env (Envelope) incorporation into the viral membrane which still is poorly understood despite some progress [49].

Obtaining a high density of virions or VLPs on grids increases throughput, since acquisition areas can be more readily clustered to permit parallel acquisition of tilt-series using beam image shift [50]. Additionally, each tilt-series contains many particles of interest for STA. Given the advantages of this approach, numerous studies on SARS-CoV-2 and other high-profile targets such as M1 of influenza-A have been conducted in recent years [51–56]. However, careful attention must be given to the effects of purification and concentration on virus structure and morphology. It is good practice to verify the authenticity of high-resolution findings with more native systems, more gentle purification protocols and different cell lines. In studying SARS-CoV-2, it was observed that fixation or inactivation, purification and concentration of the virus resulted in deviations in morphology and a decreased abundance of the prefusion state of the S-trimer when compared with viruses released from infected cells grown on grids [28,51,53]. Furthermore, in-cell analysis reveals a distinct lack of the post-fusion state, showing that its abundance in purified specimens is artefactual [28]. It is clear that despite the advantages of using purified specimens, this approach is susceptible to artefacts arising from the harsh treatment required for isolation and concentration. Even though many observations from studies of purified specimens are referred to as ‘*in situ*', we suggest that use of this term without the aforementioned diligence may lead to confusion. Furthermore, it is more likely that truly native observations are achieved by alternative methods which study viral infection processes inside cells, of which discussion will follow. Where possible, both approaches should be used to allow high-resolution structures from purified samples to be fitted within lower resolution maps obtained from cellular tomograms.

## Cryo-ET of virus–host factor interactions and membrane fusion

In early studies of HIV-1 immature particles, an unidentified density was observed in subtomogram averages of the Gag lattice, appearing at the top of the six-helix bundle (6HB) [39,40,57]. Previously overlooked as a symmetry artefact or explained as an ion cluster, only recently was this density understood to correspond to inositol hexakisphosphate (IP6), which serves as a critical cofactor for virus replication [58]. Through biochemical and crystallographic studies, IP6 was shown to be locally enriched in both the immature Gag lattice and the mature capsid lattice, serving to stabilise them through binding a series of basic residues in each [58–60]. Cryo-ET STA has since demonstrated that IP6 binding is a conserved feature of lentivirus assembly and a recent preprint manuscript shows an appearance of IP6-like density in a human endogenous betaretrovirus [61,62]. A recent study used cryo-ET to assess capsid morphologies under a range of conditions to conclude that the binding of IP6 by HIV-1 is of particular importance for the mature capsid integrity and that the immature lattice may bind IP6 in order to locally enrich it for its role in maturation [63–65]. In light of this, further study by cryo-ET STA has now suggested an additional binding site for IP6 within the mature capsid lattice in line with previous crystal structures and also compared IP6 binding to capsid pentamers versus hexamers [58,66,67]. An understanding of how IP6 stabilises the capsid lattice provides rationale for the prerequisite high salt concentrations required for formation of helical CA tubes which have previously been used for high-resolution structure determination [35,68,69]. Curiously, high-resolution subtomogram averages of bacterially expressed Gag lattices show the presence of a density in a similar position to that of IP6 despite it being lacking in prokaryotes, suggesting that other small molecules may also bind this region which may be a useful tool for future studies [41].

Multiple other host interactors of the HIV-1 capsid have been identified such as Cyclophilin A (CypA), nucleoporins (NUPs), CPSF6, MxB and Trim5α [70]. Trim5α, studied using a TRIM5α-TRIM21 chimera construct (TRIM5-21R), has long been shown to form hexagonal lattices on the surface of HIV-1 capsids, but STA of HIV-1 CA tubes in the presence of TRIM5α has since revealed the nature of this lattice [71–73]. TRIM5α has also previously been observed to disrupt the HIV-1 capsid at the inter-hexamer interfaces [74]. Additionally, binding of CypA (a host dependency factor) to native HIV-1 cores has been investigated via a novel approach for probing virus–host factor interactions within purified enveloped viruses by cryo-ET, adding to key studies using helical processing [66,68,75]. Here, the use of pore-forming perfringolysin O (PFO) treatment permits access of exogenous factors to the structures contained within the viral envelope and may be of value for further studies [66]. Despite such innovations, the study of virus–host factor interactions by cryo-ET remains limited and challenging, due to factors such as the small size of host factors (e.g. CypA), heterogeneity of binding, or the intrinsically disordered nature of others (e.g. CPSF6 and NUPs). Maturation of this field will strengthen our understanding of the processes which underpin a variety of viral replication cycles.

Cryo-ET has also been valuable in providing insight into the membrane fusion events of many viruses [76–85]. Such studies frequently rely on virus-liposome fusion events captured by cryo-ET in order to understand the virus–host fusion process and are commonly performed in a time-lapse, in which samples are vitrified at multiple time points [76–80,83,84]. Segmentation of acquired tomograms is especially helpful in aiding detailed and vivid visualisation of membrane fusion events [77,80,86].

## In-cell analysis of viral infection processes

Perhaps the most exciting avenue for the study of viral infection is using cryo-ET to unveil infection processes within their native environments. Unlike isolated viruses, the thickness of cells typically prevents access of cryo-ET to the infection occurring within. However, certain processes can sometimes be visualised without the need for sample thinning methods such as cryo-FIB/SEM. Indeed, cryo-ET has been able to access viral infection processes in selected systems for many years. More than a decade ago, Jun et al. [87] directly visualised the early stages of HIV-1 infection at the cell periphery using innovative correlative live-cell microscopy, cryo-fluorescence microscopy and cryo-ET. Later, Dai et al. [88] were able to observe phage assembly within marine cyanobacteria owing to the small size of the bacterial cells and their relative simplicity. Almost ten years later, the same group visualised aspects of chikungunya virus infection in human cells by looking at peripheral processes relevant to the final stages of alphavirus assembly. Twelve different intermediate viral structures were resolved at the plasma membrane, giving a detailed view of the process of virus assembly and providing insights into reorganisation of intermediates into the double-layered icosahedral particle observed upon budding [89]. Recently, further exploitation of electron-transparent regions of infected cells has permitted cryo-ET STA of alphavirus replication complexes and vaccinia virus maturation, as well as assembly and release of measles virus (MeV), respiratory syncytial virus (RSV) and SARS-CoV-2 [28,90–94]. Furthermore, vaccine action can be assessed by this method, as demonstrated in recent work showing ChAdOx1 nCoV-19 vaccine action by cryo-ET STA [95,96]. Clearly, cryo-ET is suitable for studying a range of processes at the cell periphery and for visualising the changes in morphology of infected cells [28,97].

However, in order to access the rest of infected cells, sample thinning methods are required to yield high-resolution insight into viral replication cycles. The cryo-FIB/SEM to cryo-ET workflow allows the process of viral assembly to be studied for viruses which, unlike chikungunya, display their assembly intermediates deeper within the infected cell. Recent studies of orthoreovirus and rotavirus have demonstrated the power of this approach to identify previously unseen assembly intermediates and visualise them to subnanometer resolutions [98,99]. Using this workflow, the investigation of SARS-CoV-2 infection by multiple groups during the COVID-19 pandemic provided a wealth of unique observations and has further generated as many questions as answers [28,100,101]. A combination of serial cryo-FIB/SEM and SXT provided observations of numerous cytopathies inside infected cells and the presence of mitochondrial defects, while also aiding speculation over the steps of viral assembly and egress [28]. This followed elucidation of a molecular pore complex spanning the double membrane of coronavirus replication compartments, using murine hepatitis virus (MHV) as a model [101]. This molecular pore is hypothesised to facilitate the release of viral RNA and mRNA from the replication compartments into the cytosol and nsp3 was suspected to be a pore constituent. Modified MHV pores containing nsp3-EGFP fusion display additional density corresponding to the EGFP moiety, confirming nsp3 as a pore constituent and Nsp3–nsp4 have since been shown to be sufficient for pore formation in a recent preprint showing improved resolution of the pore complex [101,102].

In recent years, the study of bacteriophage infection has also been aided by in-cell cryo-ET. Observation of microcompartments formed during phage 201ɸ2-1 infection in *Pseudomonas chlororaphis* have since been followed up by high-resolution analysis which confirmed that this is a conserved infection strategy between other jumbo phages [103,104]. The bacteriophage nuclear shell is believed to physically occlude the phage genome from DNA-targeting host restriction systems such as CRISPR–Cas. The role of PhuZ filaments in trafficking capsids to the phage nucleus for DNA packaging was investigated using cryo-ET following cryo-FIB/SEM and revealed capsids trapped along mutant PhuZ filaments [105]. This provides the first example of cargo trafficking in bacteria and indicates that these mechanisms are common to viral infection across different domains of life.

**Figure 2.**
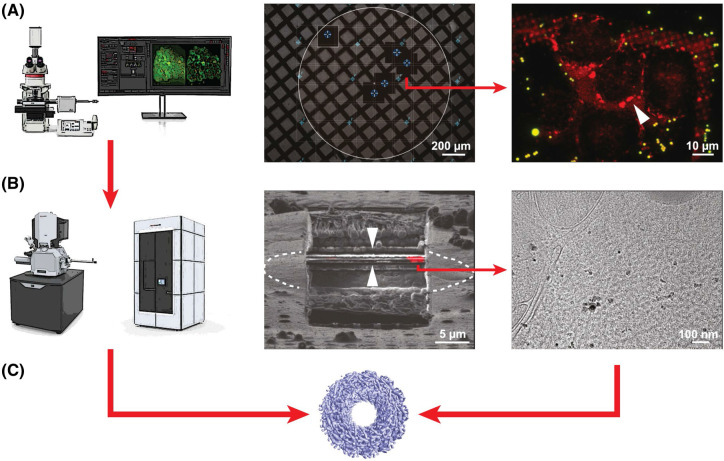
Example of correlative workflow for in-cell tomography. (**A**) cryo-CLEM permits brightfield (left) and fluorescent visualisation (right) of the sample in order to identify sites for cryo-FIB/SEM. (**B**) The fluorescent signal guides the milling process, such that the lamellae (left image) ultimately contain regions of interest, at which tilt series may be acquired (right image). (**C**) The viral structures contained within the subsequent tomograms can then be solved to subnanometer resolutions by subtomogram averaging. Figure adapted from Zhang et al. [106].

Cryo-CLEM is a powerful addition to this workflow, allowing targeting regions of interest to be processed by cryo-FIB/SEM for high-resolution cryo-ET. In a recent study of mumps virus (MuV) infection, Zhang *et al.* [106] used fluorescently labelled G3BP1 to locate stress granules, which had been observed to localise in proximity to viral replication factories. The fluorescent signal provided a guide for the milling process during cryo-FIB/SEM. MuV nucleocapsids were identified displaying different morphologies of filaments and it was observed that their abundance increased 6 h after induction of oxidative stress conditions. Analysis of these straight filaments by in-cell STA generated a 6.5 Å resolution map, which revealed additional density when compared with an *ex-situ* map of isolated filaments. This density is believed to correspond to an intrinsically disordered region of the nucleocapsid protein which becomes ordered upon binding of phosphoprotein (P), serving as a bridge to replication machinery. The power of analysing *in situ* viral structures inside infected cells using a correlative workflow, as opposed to purified isolates, is illustrated in Figure 2.

## Outlook for cryo-ET to study viral infection

Advances in cryo-ET and complementary methods will fuel progress towards an in-depth understanding of viral infection in the coming years. It can be expected that viral assembly intermediates for a greater range of viruses will be characterised, increasing our knowledge of how they cause human disease. This work will lay foundations for rapid responses to emerging pathogens in the future.

Developments in microscope hardware and image processing methods are enabling ever-higher resolution structures to be determined by cryo-ET STA with ever-increasing throughput. Advanced sample preparation methods such as cryo-FIB/SEM are opening up previously uncharted cellular landscapes to cryo-ET. To better understand processes occurring in the crowded cellular environment, cryo-CLEM is likely to become a vital tool in dissecting viral infection processes. Innovative labelling strategies will be needed to realise the full potential of this method, since establishment of replication-competent engineered virus for expression of fluorescent critical viral components is challenging.

An interesting area of active development is the use of phase plates to allow high-contrast imaging without applying defocus [82,88]. While the current ‘Volta' phase plate has limited use for high-resolution averaging, new laser phase plates which harness electron-light interactions to generate contrast in an obstruction-free manner may be of great value to cryo-EM and cryo-ET [107]. Furthermore, emergence of chromatic aberration (C_c_) correction shows potential for increasing signal by making use of inelastically scattered electrons which are currently either eliminated from image formation or contribute to noise [108,109].

Alongside hardware development, software innovations are enhancing the power of collected data [110]. Areas of intensive development include machine-learning approaches to particle picking and segmentation of surfaces [111–113]. Additionally, improved software for automated tilt-series alignment (and refinement) will further improve tomogram quality and consequently the resolutions of subtomogram averages [18,19]. Further developments in addressing sample heterogeneity and conformational landscapes are important, with recent developments providing unbiased analysis of heterogeneity and its contextualisation within *in situ* cellular data [114].

Aside from cryo-ET, developments in other potential high-resolution imaging techniques such as electron ptychography should be monitored closely, although much progress is still to be made in these areas in order to reach subnanometer resolutions for biological samples [115]. Ultimately, through a combination of the approaches presented in this review, it is hoped that processes such as assembly and egress will be monitored in real time and characterised with structural snapshots of each stage and at multiple scales.

## Perspectives

- With cryo-ET and complementary methods gaining traction in recent years, it is expected that viral replication and infection processes for a greater range of viruses will be characterised, increasing our understanding of how they cause human disease and aiding vaccine and anti-viral drug development.
- Improvement in microscope hardware and image processing methods are enabling higher resolution structures to be determined with increasing throughput by cryo-ET STA. Advanced sample preparation methods such as cryo-FIB/SEM and cryo-CLEM are opening up previously uncharted cellular landscapes to cryo-ET.
- Further developments to increase signal to noise ratio (such as near-perfect direct detector, C_C_ correction and laser phase plate) and innovations in time-resolved approaches, will allow us to monitor virus infection processes such as assembly and egress in real time and characterise high-resolution structural snapshots of each stage.

## Open Access

Open access for this article was enabled by the participation of University of Oxford in an all-inclusive *Read & Publish* agreement with Portland Press and the Biochemical Society under a transformative agreement with JISC.

## Abbreviations

C_c_: chromatic aberration
CLEM: correlative light electron microscopy
IP6: hexakisphosphate
MA: matrix
MHV: murine hepatitis virus
NUPs: nucleoporins
RNP: ribonucleoprotein complex
SXT: soft x-ray tomography
VLPs: virus-like particles
